# PFAS Compounds Display Distinct Toxicological Effects in *Drosophila melanogaster*, Reflected by Reduced Viability and Impaired Neuronal Function

**DOI:** 10.3390/biom16040557

**Published:** 2026-04-09

**Authors:** Klara Luedtke, Cristian Blanco Rocha, Magdalena Svensson, Ann-Christin Brorsson

**Affiliations:** Department of Physics, Chemistry, and Biology, Linköping University, 581 83 Linköping, Swedenmagdalena.svensson@liu.se (M.S.)

**Keywords:** per- and polyfluoroalkyl substances (PFAS), perfluorooctanoic acid (PFOA), perfluorononanoic acid (PFNA), *Drosophila melanogaster*, acetylcholinesterase (AChE), neurotoxicity, survival assay, toxicological profiling

## Abstract

Per- and polyfluoroalkyl substances (PFAS) are environmentally persistent chemicals associated with a wide range of adverse health effects, yet individual PFAS compounds may exert distinct toxicological mechanisms. In this study, we investigate the toxic effects of perfluorooctanoic acid (PFOA) and perfluorononanoic acid (PFNA) in *Drosophila melanogaster* using survival assays and measurements of acetylcholinesterase (AChE) activity as indicators of systematic toxicity and neurotoxicity, respectively. Male flies were exposed to PFOA and PFNA under different feeding conditions, concentrations, and temperatures. Both compounds reduced fly viability and impaired neuronal function, but with markedly different toxicological profiles. PFNA caused a pronounced, concentration-dependent reduction in lifespan under all tested conditions, indicating a strong systemic toxicity. In contrast, PFOA exerted a comparatively weaker effect on survival but induced a more pronounced reduction in AChE activity, consistent with enhanced neurotoxicity. PFOA-induced neurotoxicity in *Drosophila* may represent early molecular events that predispose neurons to degeneration, contributing to conditions such as dementia. Together, these findings demonstrate that structurally similar PFAS compounds can induce distinct toxicological outcomes and highlight the importance of evaluating individual PFAS using complementary assays. Moreover, this study underscores the utility of *Drosophila melanogaster* as a sensitive and mechanistically informative model for dissecting compound-specific PFAS toxicity.

## 1. Introduction

**Figure 1:**
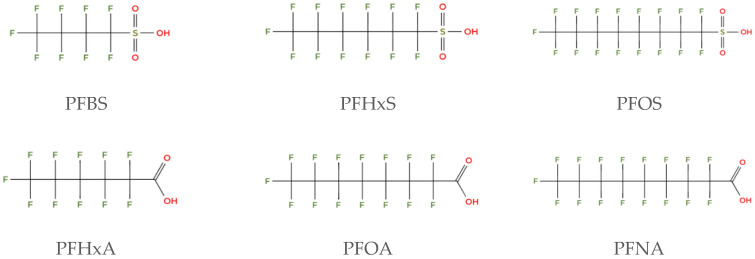
Chemical structure of selected per- and polyfluoroalkyl substances (PFAS): perfluorobutanesulfonic acid (PFBS), perfluorohexanosulfonic (PFHxS), perfluorooctanesulfonic acid (PFOS), perfluorohexanoic acid (PFHxA), perfluorooctanoic acid (PFOA), and perfluorononanoic acid (PFNA).

Per- and polyfluoroalkyl substances (PFAS) are a class of highly fluorinated chemicals with over 10,000 identified substances (Figure 1). They are known for their ability to repel water, oil, and grease, and to function as a surfactant which makes them useful in a wide range of consumer and industrial products. PFAS are often referred to as “forever chemicals” because they break down very slowly in the environment and can accumulate in the bodies of humans and animals over time.

PFAS contamination affects multiple environmental media, including groundwater, surface water, soil, and air. Their strong carbon–fluorine bonds contribute to their persistence and bioaccumulation, making them a long-term environmental concern [1,2,3,4]. Today PFAS are found throughout the environment, and studies have shown that approximately 99% of the human population, including fetuses, carry detectable levels of these chemicals in their blood [5,6]. Ongoing research and regulatory efforts are crucial to address the impact of these persistent pollutants on human health and the environment. Exposure to PFAS has been linked to several adverse health outcomes, such as increased cholesterol levels, liver and kidney damage, immune system suppression, low birth weight, impaired vaccine response, cardiometabolic disorders, and disruption of endocrine and reproductive systems [7,8]. Some PFAS can cross the blood–brain barrier and accumulate in the central nervous system, potentially leading to neurotoxic effects [9,10]. Studies have reported associations between PFAS exposure and neurotoxic mechanisms, including neurotransmitter disruption, oxidative stress, mitochondrial dysfunction, calcium dysregulation and neuroinflammation [9,10]. Emerging epidemiological and experimental evidence also suggest a possible link between PFAS exposure and neurodegenerative diseases, such as Alzheimer’s and Parkinson’s, although current human data remain limited [6,11,12]. Furthermore, endocrine-disruption effects, including altered steroidogenesis and thyroid hormone signaling, have been specifically associated with exposure to perfluorooctanoic acid (PFOA). In contrast, reduction in birth weight and related fetal growth metrics have been consistently linked to exposure to perfluorononanoic acid (PFNA) [13,14]. By studying PFAS toxicity to elucidate the mechanisms by which these persistent chemicals affect human health and the environment, we will be better equipped to protect both people and ecosystems from their harmful effects.

In this study, the toxic effects of the PFAS compounds PFOA and PFNA were evaluated in *Drosophila melanogaster* (fruit fly) by survival assays and acetylcholinesterase (AChE) activity. *Drosophila melanogaster* is a widely used model organism for investigating the adverse effects of toxic compounds and proteins [15,16,17,18,19]. Compounds are commonly administered to flies by incorporating them into the diet, while targeted protein expression is achieved through the Gal4/UAS genetic system [20]. The fruit fly shares approximately 60% of its genes with humans, and about 75% of human disease-related genes have homologs in *Drosophila* [21,22]. It has a central brain, peripheral sensory neurons, and neuromuscular junctions that resemble human systems. Moreover, many metabolic pathways and physiological functions are conserved between *Drosophila* and mammals, making it a powerful tool for extrapolating experimental findings to human biology [23,24,25].

Using survival assays to assess the toxic effects of a compound in *Drosophila melanogaster* provides a broad overview of its impact on the organism. A reduced lifespan in drug exposed flies suggests that the compound may interfere with essential biological processes critical for survival [26]. In contrast, using AChE activity as a toxicological readout provides a more targeted assessment of a compound’s effect. A reduction in AChE activity indicates neurotoxicity, as this enzyme plays a critical role in maintaining proper neuronal function by regulating neurotransmitter levels [27,28].

The present study identified several key factors influencing the toxic effects of PFOA and PFNA in *Drosophila*, including compound concentration, alkyl chain length, ambient temperature, and food composition. Specifically, we found that both PFOA and PFNA exert toxic effects in *Drosophila melanogaster*, as evidenced by reduced lifespan and decreased acetylcholinesterase (AChE) activity. However, PFNA exhibited greater toxicity in survival assays, indicating a stronger impact on overall viability. In contrast, PFOA showed a more pronounced reduction in acetylcholinesterase activity, suggesting a higher degree of neurotoxicity. These distinct toxicological profiles underscore the utility of *Drosophila melanogaster* as a model organism for dissecting the mechanisms underlying chemical toxicity and for identifying potential protective strategies. Furthermore, the findings suggest that the diverse health issues associated with PFAS exposure in humans may be driven by different components within the PFAS mixture. Therefore, understanding the specific toxic mechanisms of individual PFAS compounds is crucial for developing effective strategies to mitigate PFAS-induced health effects.

## 2. Materials and Methods

### 2.1. Drosophila Line

This study utilized the Dahomey *Drosophila* line, with flies maintained at either 25 °C or 29 °C under 65% humidity and a 12-h light/12-h dark cycle. The flies were transferred every 2–3 days into new vials that contained either standard food (1.01% *w*/*v* agar, 6.82% *v*/*v* molasses, 6.82% *w*/*v* maize, 2.81% *w*/*v* yeast, 0.15% *w*/*v* nipagin, 1.50% *v*/*v* ethanol, and 0.55% *v*/*v* propanoic acid) ± PFAS or filter paper soaked in 10% sucrose ± PFAS. In the fly experiments, male flies were selected and maintained on standard food for one day prior to PFAS exposure. The rationale for using males is to avoid the food becoming sticky from egg-laying by females, which increases the risk of flies becoming trapped in the food. In all experiments, control flies were included and treated with either standard food or 10% sucrose without PFAS.

### 2.2. Longevity Assay

Flies were divided in groups of 20 and transferred every 2–3 days into new vials with standard food ± PFAS or 10% sucrose ± PFAS. Simultaneously, the number of dead and live flies was counted, and the process was repeated until all flies had died. Kaplan–Meier survival curves [29] were generated using GraphPad Prism software 10 [30] and longevity statistics were analyzed.

### 2.3. Acetylcholinesterase Activity Assay

Acetylcholinesterase (AChE) activity was measured using the Sigma-Aldrich assay kit (CS0003) (Sigma-Aldrich, St. Louis, MO, USA). Flies were decapitated and five heads or five bodies were homogenized in 60 µL or 120 µL of assay buffer, respectively. The homogenates were centrifuged at 12,000× *g* (heads) or 20,000× *g* (bodies) for 5 min. The resulting supernatants were collected and diluted 1:5 in assay buffer. A volume of 50 µL of the diluted supernatant was added to a 96-well plate along with 50 µL of substrate mix. Samples were analyzed using the Tecan Infinite M1000 Pro plate reader (Tecan Group Ltd., Männedorf, Switzerland) by recording absorbance at 412 nm for 10 min. Two fly homogenates were analyzed at 25 °C and three at 29 °C, with all samples run in duplicate.

### 2.4. Chemicals and Sample Preparation

The chemicals used in this study included perfluorooctanoic acid (PFOA, CAS No. 335-67-1), perfluorononanoic acid (PFNA, CAS No. 375-95-1), octanoic acid (OA, CAS No. 124-07-2), and nonanoic acid (NA, CAS No. 112-05-0), all purchased from Sigma-Aldrich. PFOA and PFNA were dissolved in Milli-Q water to prepare 2 mM stock solutions, which were subsequently diluted to the desired concentrations. For OA and NA, 20 mM stock solutions were prepared by diluting the concentrated acids in acetone. Calculated aliquots of these stock solutions were transferred to 1 mL vials, and the acetone was allowed to evaporate overnight. Thereafter, 0.5 mL of 10% sucrose solution was added to each vial, resulting in final concentrations of 0.5 mM, 1 mM, 3 mM and 5 mM of OA or NA used in the study.

### 2.5. Statistical Analysis

The data was analyzed using GraphPad Software 10. Kaplan–Meier survival curves were generated using GraphPad Prism software 10 [30].

## 3. Results

### 3.1. Concentration Dependence of PFOA/PFNA Induced Mortality

**Figure 2:**
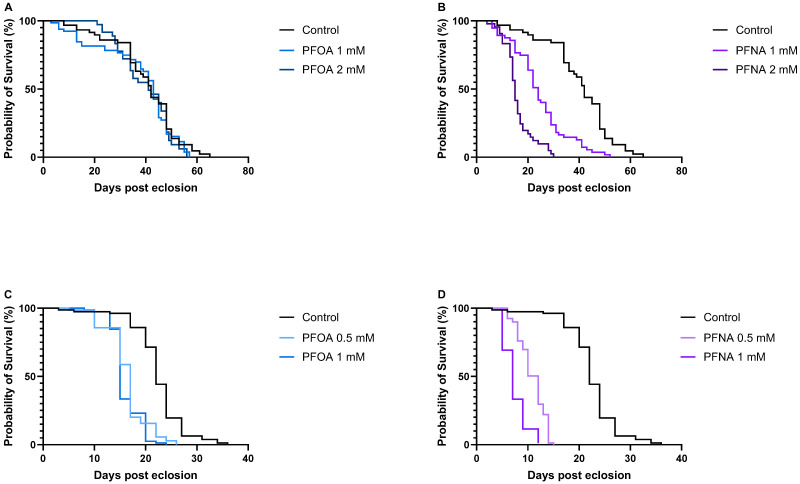
Survival of flies exposed to increasing concentrations of PFOA and PFNA, where the median survival time (MST) is defined as the time point corresponding to 50% survival. (**A**) Flies exposed to PFOA on standard food (*n* = 47, *n* = 59 and *n* = 34 for control, 1 mM, and 2 mM, respectively). (**B**) Flies exposed to PFNA on standard food (*n* = 47, *n* = 55 and *n* = 41 for control, 1 mM, and 2 mM, respectively). (**C**) Flies exposed to PFOA on 10% sucrose (*n* = 77, *n* = 70 and *n* = 78 for control, 0.5 mM, and 1 mM, respectively). (**D**) Flies exposed to PFNA on 10% sucrose (*n* = 77, *n* = 78, and *n* = 77 for control, 0.5 mM, and 1 mM, respectively). The control group in (**A**,**B**) consisted of flies exposed only to standard food, and the control group in (**C**,**D**) consisted of flies exposed only to 10% sucrose.

**Figure 3:**
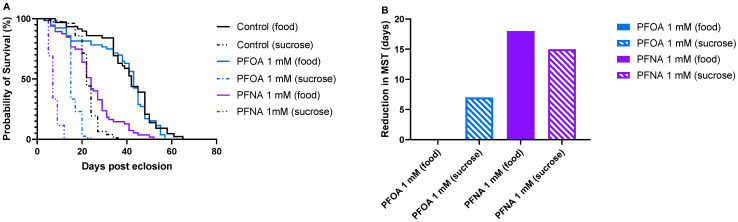
Survival of control, PFOA- or PFNA-exposed flies fed on standard food or 10% sucrose. (**A**) Control flies and flies exposed to 1 mM PFOA or 1 mM PFNA at the two different feeding conditions. (**B**) Diagram showing the reduction in median survival between control flies and PFOA/PFNA-exposed flies treated with standard food or with 10% sucrose.

To investigate the mortality effects of PFOA and PFNA in *Drosophila melanogaster*, survival assays were conducted in which flies were exposed to varying concentrations of the PFAS molecules while fed standard food or 10% sucrose. Flies maintained on standard food were exposed to 1 mM and 2 mM PFOA (Figure 2A) or 1 mM and 2 mM PFNA (Figure 2B), while flies fed with 10% sucrose were exposed to 0.5 mM and 1 mM PFOA (Figure 2C) or 0.5 mM and 1 mM PFNA (Figure 2D). All survival experiments were performed at 25 °C. Median survival time (MST), defined as the day when 50% of the flies had died, was used to evaluate toxicity under each condition. The MST for food-treated and sucrose-treated control flies were 42 and 22 days, respectively, revealing that flies maintained on sucrose exhibited reduced overall health compared to those on standard food. Further analysis showed that all PFNA-exposed flies had significantly reduced longevity compared to their respective controls. Food-treated flies exposed to 1 mM and 2 mM PFNA had a reduction in median survival time of 18 (*p* < 0.0001), and 27 (*p* < 0.0001) days, respectively, where the difference in MST between 1 mM and 2 mM PFNA-exposed flies was 9 days (*p* < 0.0001). Sucrose-treated flies exposed to 0.5 mM and 1 mM PFNA had a reduction in MST of 10 (*p* < 0.0001), and 15 (*p* < 0.0001) days, respectively, where the difference in MST between 0.5 mM and 1 mM PFNA exposed flies was 5 days (*p* < 0.0001). These reductions in lifespan clearly demonstrate that PFNA is toxic to flies and that its toxicity occurs in a dose-dependent manner. Interestingly, PFOA displayed a different toxic behavior compared to PFNA. No reduced longevity was observed when flies were exposed to 1 mM or 2 mM PFOA on standard food. In contrast, sucrose-fed flies exposed to 0.5 mM and 1 mM PFOA showed a significant reduction in median survival time (MST) by 5 days (*p* < 0.0001) and 7 days (*p* < 0.0001), respectively, indicating a toxic response under these conditions. However, the 2-day difference in MST between 0.5 mM and 1 mM PFOA exposed flies was not statistically significant, suggesting that increasing the PFOA concentration did not further enhance its toxic effect on fly survival. Comparison of survival curves between food-treated and sucrose-treated control, 1 mM PFOA-exposed and 1 mM PFNA-exposed flies (Figure 3A) showed a considerable reduction in MST between control and PFNA-exposed flies both on standard food and 10% sucrose, revealing that PFNA induces mortality under both feeding conditions (Figure 3B). In contrast, for PFOA-exposed flies, a decrease in MST was observed only in those fed with 10% sucrose, suggesting that PFOA exerts its mortality effects more readily under sucrose feeding conditions than on standard food (Figure 3B).

### 3.2. Alkyl Chain Length Dependence of PFOA/PFNA Induced Mortality

**Figure 4:**
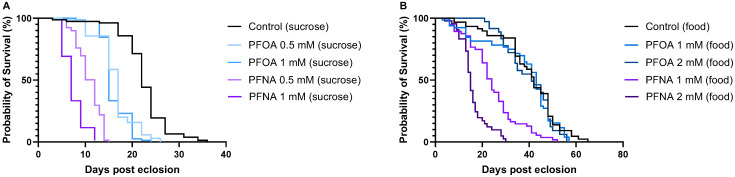
Survival of flies exposed to PFOA or PFNA when treated with (**A**) 10% sucrose and (**B**) standard food. Flies receiving only 10% sucrose or standard food served as controls.

Analysis of survival curves for flies exposed to varying concentrations of PFOA and PFNA under the two feeding conditions, 10% sucrose (Figure 4A) and standard food (Figure 4B), revealed that PFNA consistently caused the most pronounced reduction in MST compared to PFOA. In sucrose-treated flies, MST dropped to 12 and 7 days for 0.5 mM and 1 mM PFNA exposure, respectively, whereas PFOA exposure at the same concentrations resulted in MST of 17 and 15 days. Similarly, in food-treated flies, MST was 24 and 15 days for 1 mM and 2 mM PFNA, compared to 43 and 41 days for 1 mM and 2 mM PFOA. These findings show that alkyl chain length affects fly mortality, with longer PFNA (9-carbon chain) exhibiting higher mortality than shorter PFOA (8-carbon chain).

### 3.3. PFOA/PFNA Exposure Leads to Impaired Neuronal Function in the Fly Brain

**Figure 5:**
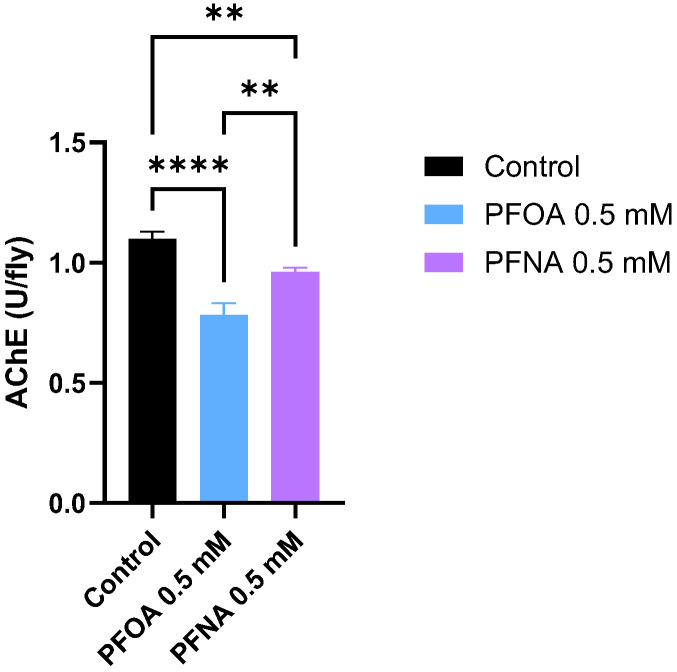
Acetylcholinesterase activity in flies exposed to PFOA and PFNA. Flies were treated with PFAS compounds in combination with 10% sucrose at 25 °C for 7 days; control flies received only 10% sucrose. Data are presented as mean ± SD. Statistical significance was assessed using one-way ANOVA followed by Tukey’s test (** *p* < 0.01; **** *p* < 0.0001).

To determine whether PFOA and PFNA exposure damages neuronal function in flies, acetylcholinesterase (AChE) activity was measured in the heads of flies exposed to PFOA or PFNA. AChE plays a critical role in neurotransmission by degrading the neurotransmitter acetylcholine (ACh). Rapid breakdown of ACh is essential for neurons to reset and process new signals efficiently. Reduced AChE activity can lead to ACh accumulation, resulting in neurotoxicity. In this assay, flies were exposed to 0.5 mM PFOA or PFNA in 10% sucrose at 25 °C for 7 days, after which AChE activity was measured in fly heads calculated as U/fly (where 1 U = 1 µmol of substrate converted per minute). The result showed that PFAS exposure impairs neuronal function in the fly brain, as evidenced by a significant reduction in AChE activity in both PFOA- and PFNA-exposed flies compared to control. AChE activity was measured at 0.78 U/fly in PFOA-exposed flies, 0.96 U/fly in PFNA-exposed flies, and 1.10 U/fly in control flies (Figure 5). Notably, AChE activity was significantly lower in PFOA-exposed flies compared to PFNA-exposed flies, suggesting that PFOA exerts a stronger neurotoxic effect in the fly brain.

### 3.4. Temperature Has Limited Influence on PFOA/PFNA Induced Mortality

**Figure 6:**
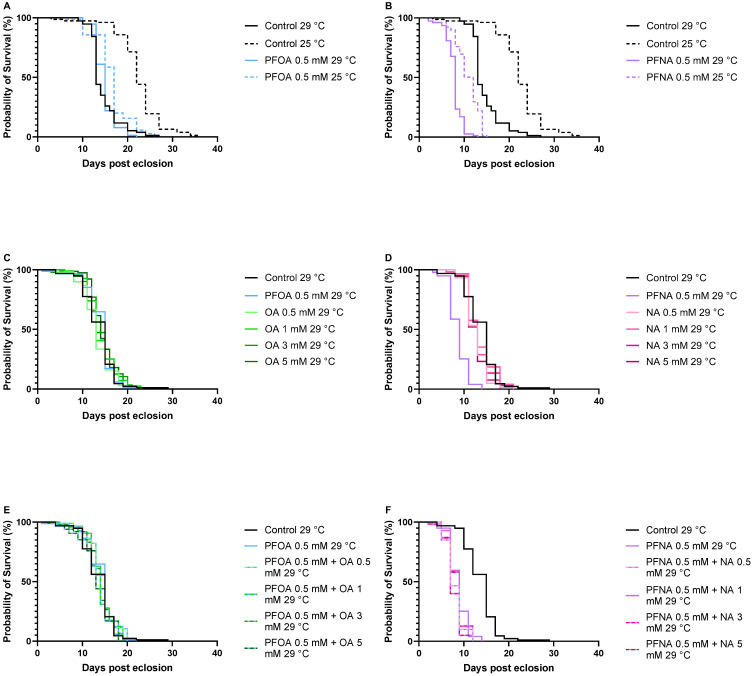
Survival of (**A**) flies exposed to 0.5 mM PFOA at 29 °C (*n* = 77) and 25 °C (*n* = 70), (**B**) flies exposed to 0.5 mM PFNA at 29 °C (*n* = 77) and 25 °C (*n* = 78), (**C**) flies exposed to 0.5 mM PFOA and 0.5 mM, 1 mM, 3 mM, and 5 mM OA at 29 °C (*n* = 87, *n* = 99, *n* = 96, *n* = 90 and *n* = 78, respectively), (**D**) flies exposed to 0.5 mM PFNA and 0.5 mM, 1 mM, 3 mM, and 5 mM NA at 29 °C (*n* = 99, *n* = 60, *n* = 53, *n* = 59 and *n* = 53, respectively), (**E**) flies exposed to 0.5 mM PFOA in combination with 0.5 mM, 1 mM, 3 mM, and 5 mM NA, respectively at 29 °C (*n* = 96, *n* = 97, *n* = 93, *n* = 100), (**F**) flies exposed to 0.5 mM PFNA in combination with 0.5 mM, 1 mM, 3 mM, and 5 mM NA, respectively, at 29 °C (*n* = 60, *n* = 57, *n* = 60, *n* =62). Flies were exposed to PFAS compounds in 10% sucrose, either alone or in combination with the corresponding fatty acid. Flies receiving only 10% sucrose served as controls (*n* = 77 for both controls at 29 °C and 25 °C in (**A**,**B**) and *n* = 94 for the control at 29 °C in (**C**–**F**)).

Survival assays using 10% sucrose were conducted at 29 °C and compared to the survival data at 25 °C to assess the temperature dependence of PFOA and PFNA toxicity. For control flies, median survival time (MST) was significantly lower at 29 °C (13 days) compared to 25 °C (22 days) (*p* < 0.0001), indicating that higher temperatures reduce fly viability (Figure 6A,B). Interestingly, this temperature effect was not as prominent in PFOA-exposed flies: MST were 15 and 17 days for PFOA at 29 °C and 25 °C (*p* < 0.0001), respectively (Figure 6A). The temperature effect for PFNA-exposed flies was somewhat larger compared to PFOA: MST were 8 and 12 days for PFNA at 29 °C and 25 °C (*p* < 0.0001), respectively, (Figure 6B), but still not as large as for the control flies. These data suggest that the effect of PFOA and PFNA on fly viability is only slightly influenced by temperature, with PFNA being somewhat more affected than PFOA. Notably, data showed that PFNA exerted a significant toxic effect at both 25 °C and 29 °C, reducing median survival time by 10 (*p* < 0.0001) and 5 (*p* < 0.0001) days, respectively, compared to controls. In contrast, the effect of PFOA on fly viability was evident only at 25 °C, with an MST reduction of 5 days (*p* < 0.0001), while no significant reduction in MST was observed at 29 °C. Thus, unlike PFNA, PFOA does not reduce the lifespan of the flies at 29 °C. A possible explanation is that at 29 °C, the factors that shorten lifespan overshadow PFOA toxicity, making the presence of the PFOA molecule irrelevant to fly mortality at 29 °C compared to 25 °C.

In the survival assay at 29 °C, different concentrations of the corresponding non-fluorinated acids to PFOA and PFNA, octanoic acid (OA) and nonanoic acid (NA), respectively, were included to investigate the impact of these compounds on the life span (Figure 6C,D). No significant reduction in MST was observed at any concentration of OA or NA compared to control. MST values for OA at 0.5 mM, 1 mM, 3 mM, and 5 mM were 13, 13, 13, and 14 days, respectively, while MST for NA remained 13 days across all concentrations. A survival assay was also conducted for flies exposed to PFOA and PFNA in the presence of varying concentrations of the corresponding non-fluorinated acids (Figure 6E,F). The results show that the toxicity of PFOA and PFNA is not affected by the presence of OA or NA in the diet, even when the lipid concentration is six times higher than that of the PFAS. MST for the PFOA-exposed flies ranged from 13 to 15 days, whereas PFNA exposure resulted in an MST of 7 to 9 days.

### 3.5. PFOA Exposure Leads to Impaired AChE Activity at 29 °C

**Figure 7:**
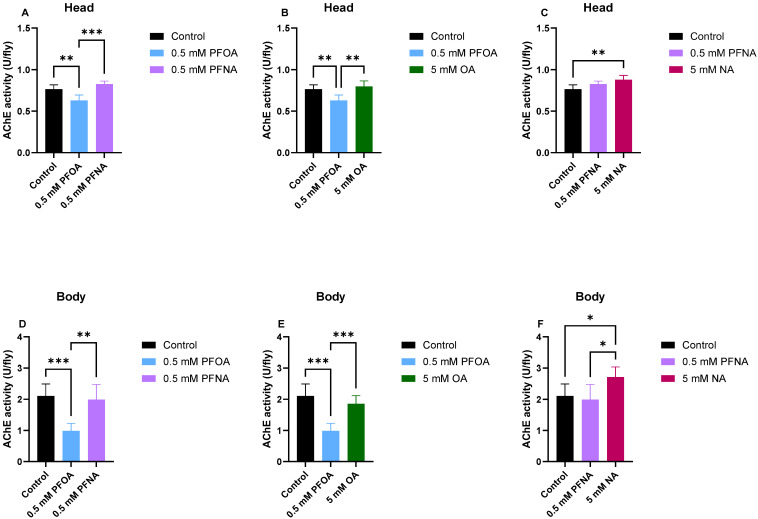
Acetylcholinesterase activity of PFOA-, PFNA-, OA-, and NA-exposed flies in the head (**A**–**C**) or body (**D**–**F**) of the flies. The compounds were exposed to the flies in combination with 10% sucrose at 29 °C for 5 days. Control flies received only 10% sucrose. Values are expressed as means ± SD. Significance was determined by Welch’s ANOVA followed by Dunnett’s T3 test (* *p* < 0.05; ** *p* < 0.01; *** *p* < 0.001).

AChE activity analyses of flies exposed to PFOA, PFNA, OA and NA at 29 °C in 10% sucrose were then performed to elucidate the impact of these compounds on the degradation of ACh both in the head and in the body of the flies. The assay was performed after 5 days of incubation. In the fly head, a significant AChE activity reduction was detected in the PFOA flies compared to both control (*p* < 0.01) and PFNA flies (*p* < 0.001) while the AChE activity in the PFNA flies was similar to the control flies (Figure 7A). The AChE activity in the PFOA flies was also significantly reduced compared to OA flies (*p* < 0.01) while the AChE activity in the OA flies was similar to the control flies (Figure 7B). No significant difference in the AChE activity between PFNA and NA flies was observed (Figure 7C). The significant increase in AChE activity (*p* < 0.01) observed in NA-exposed flies compared with controls may indicate a beneficial effect of NA on AChE activity. Alternatively, this difference could simply reflect the altered dietary composition between the control and NA-treated groups. Data observed in the fly body analyses essentially mirrors the result from the fly heads where a significant AChE activity reduction was detected in the PFOA flies compared to both control (*p* < 0.001) and PFNA flies (*p* < 0.01) while the AChE activity in the PFNA flies was similar to the control flies (Figure 7D). The AChE activity in the PFOA flies was also significantly reduced compared to OA flies (*p* < 0.001) while the AChE activity in the OA flies was similar to the control flies (Figure 7E). A significant increase in AChE activity was observed in NA-exposed flies compared to control (*p* < 0.05) (Figure 7F) and this increase was also significant compared to PFNA flies (*p* < 0.05) reinforcing the suggestion that NA could be beneficial for AChE activity. Interestingly, PFOA caused a greater reduction in AChE activity in the body than in the head, with activity decreasing by 50% in the body compared to 20% in the head.

### 3.6. PFOA and PFNA Differ in Their Toxicological Profiles

Overall, the analyses demonstrate that both PFOA and PFNA exert adverse effects in flies. Interestingly, comparison of survival data with AChE activity measurements suggests that PFNA shows greater toxicity in survival assays, whereas PFOA has a stronger impact on AChE activity. Data also show that presence of fluorine atoms is critical for PFNA-toxicity in the survival assay, since MST for PFNA-exposed flies was significantly (*p* < 0.0001) reduced by four days compared to all tested concentrations of NA, and that presence of fluorine atoms is critical for PFOA-toxicity on AChE activity since the AChE activity in PFOA-exposed flies was significantly reduced compared with OA-exposed flies both in the head (*p* < 0.01) and in the body (*p* < 0.001). Table 1 summarizes the calculated toxicity factors (TF) for PFOA and PFNA across the different experiments. Overall, TFs were higher for PFNA than for PFOA in the survival assays, whereas in the acetylcholinesterase assay, TFs were higher for PFOA than for PFNA.

Indeed, data in Table 1 highlights that while the preliminary toxicity of PFOA is reduced AChE activity, the toxicity of PFNA does not impact the AChE activity to the same extent but rather acts on the lifespan of the fly by increasing the mortality. This suggests that the targets in the toxic mechanism of PFOA and PFNA are different, causing different phenotypical outcomes.

## 4. Discussion

PFAS are synthetic chemicals known for their extreme environmental persistence, largely due to the strength of their carbon–fluorine bonds. These compounds contaminate water, soil, and air worldwide, making human and ecological exposure nearly unavoidable [2,3,4]. PFAS not only persist in the environment but also bioaccumulate in human tissues, raising significant health concerns [5,7]. There are thousands of PFAS compounds, each with different chain structures and functional groups [2]. Exposure to PFAS has been associated with a wide range of adverse health effects, including hormonal disruption, immune system suppression, liver damage, developmental issues in infants and children, neurodegeneration, and an increased risk of certain cancers [7,8]. Understanding the toxicity of individual PFAS compounds is essential for identifying which are most harmful and how they exert their effects. Investigating their molecular mechanisms of action can guide the development of targeted interventions, such as safer chemical alternatives, effective remediation strategies, and medical treatments for exposed populations, which, among others, has been discussed previously by Yaghoobian et al. and Hu and Scott [31,32].

Our findings reveal striking differences in the toxicological profiles of PFOA and PFNA in *Drosophila melanogaster*, despite the close structural similarity of these two PFAS compounds, reinforcing that PFAS should not be treated as chemically uniform substances [33,34,35]. PFNA mortality exhibits a strong concentration dependence, as survival assays demonstrated a clear dose–response relationship: increasing PFNA concentrations progressively shortened median survival times in both food-treated and sucrose-treated flies. This pattern likely reflects the physicochemical properties of PFNA, where higher concentrations increase bioavailability and promote interactions with essential biological processes critical for survival. In contrast, no reduction in viability was observed in food-treated flies exposed to either 1 mM or 2 mM PFOA. This suggests that, unlike PFNA, nutritional components present in standard food may attenuate the impact of PFOA on survival. Conversely, sucrose-fed flies exposed to PFOA exhibited significant mortality at 0.5 mM. Notably, this adverse effect did not intensify at higher concentrations, indicating that, unlike PFNA, the lethal cellular interactions of PFOA do not scale with dose. Rather, its toxic influence appears to plateau at approximately 0.5 mM.

Survival assays at different temperatures showed that temperature significantly affects the overall viability of *Drosophila melanogaster*, but only marginally increases the toxicity of PFOA and PFNA. Control flies showed a significantly shorter median survival time at 29 °C compared to 25 °C (a reduction of 9 days), confirming that elevated temperatures impose physiological stress and shorten lifespan. In contrast, the temperature-related decrease was only 4 days for PFNA-exposed flies and 2 days for PFOA-exposed flies. This limited enhancement of additive or synergistic effects between heat stress and PFOA/PFNA exposure suggests that their toxicity acts through mechanisms already close to maximal disruption under standard conditions, leaving little scope for further exacerbation by temperature. Notably, the toxic mechanism of PFNA appears slightly more sensitive to temperature increases than that of PFOA. Alternatively, PFOA/PFNA-induced cellular impairments might, to a certain extent, overshadow temperature-related increased mortality. This pattern may indicate that the effects of PFOA and PFNA on lifespan are not highly sensitive to moderate temperature variation. Nonetheless, potential interactions between extreme heat stress and PFAS exposure remain to be explored.

Analysis of PFOA/PFNA mortality in relation to alkyl chain length showed that survival in *Drosophila melanogaster* is chain-length dependent. The longer nine-carbon PFNA consistently caused a significantly greater reduction in lifespan than the shorter eight-carbon PFOA across all tested concentrations, temperatures, and feeding conditions. This pattern suggests that mortality increases with chain length, consistent with the physicochemical properties of PFAS. Longer-chain PFAS exhibit higher hydrophobicity potentially leading to greater bioaccumulation and persistence in tissues, which likely amplifies their toxic potential. Interestingly, results from the AChE activity assays provided additional mechanistic insights that contrasted with the survival assay findings. While increased alkyl chain length was associated with greater overall toxicity in survival assays, this trend did not hold in the context of neurotoxicity. Both PFOA and PFNA significantly reduced AChE activity in fly heads at 25 °C, indicating impaired neuronal function. AChE is essential for proper neurotransmission, and its inhibition can lead to acetylcholine accumulation and synaptic dysfunction. However, PFOA induced a more substantial reduction in AChE activity across all experimental conditions: in the head at 25 °C (toxicity factor (TF) = 29%) and 29 °C (TF = 20%), as well as in the body at 29 °C (TF = 50%). In contrast, PFNA showed a detectable effect only in the head at 25 °C (TF = 13%), despite causing the most pronounced reduction in lifespan. Thus, PFNA and PFOA differ not only in overall toxicity but also in their specific neurotoxic profiles, highlighting the importance of using multiple assays to uncover distinct mechanisms of PFAS-induced toxicity. Furthermore, the observation that PFOA reduced AChE activity more strongly in the body than in the head highlights potential tissue-specific effects, which could influence the manifestation of neurotoxicity versus systemic toxicity. The observed discrepancy in the toxicological profiles of PFOA and PFNA suggests that while chain length correlates with overall systemic toxicity, specific adverse molecular interactions—such as enzyme inhibition—do likely depend on more precise interaction between the PFAS compound and the target molecule suggesting that different PFAS compounds may target distinct biological pathways.

## 5. Conclusions

In summary, both PFOA and PFNA have significant adverse effects on fruit flies, but data suggest that these compounds act through partially different toxic mechanisms. PFNA exhibited the strongest effect on survival, reducing median lifespan more than PFOA under all tested conditions. This indicates that PFNA exerts a more pronounced systemic toxicity, likely linked to its longer alkyl chain, which enhances bioaccumulation and persistence in tissues. In contrast, neurotoxicity assays revealed that PFOA caused a greater reduction in AChE activity compared to PFNA, despite its weaker impact on overall survival. This suggests that PFOA may preferentially target neuronal or neuromuscular systems, impairing cholinergic signaling more effectively than PFNA. Indeed, these findings indicate that chain length not only determines the degree of PFAS bioaccumulation and persistence but may also influence the mode of toxicity, including neurotoxic pathways. This has important implications for risk assessment, as shorter-chain PFAS are often considered safer alternatives, yet our data suggest that structural differences can lead to distinct toxicological profiles beyond simple chain-length trends. The pronounced impact of PFOA on AChE activity in *Drosophila melanogaster* suggests a clear neurotoxic effect. AChE is critical for terminating synaptic transmission by hydrolyzing acetylcholine; its inhibition leads to excessive cholinergic signaling, neuronal stress, and ultimately dysfunction. This mechanism is particularly relevant because impaired cholinergic signaling and progressive neuronal degeneration are hallmarks of several human neurodegenerative disorders, including Alzheimer’s disease, Parkinson’s disease, and amyotrophic lateral sclerosis (ALS). Therefore, PFOA-induced neurotoxicity in *Drosophila* may represent early molecular events that predispose neurons to degeneration, contributing to conditions such as dementia. This makes the *Drosophila* model of PFAS toxicity an invaluable tool for identifying PFAS compounds that may play a role in neurodegenerative disease and for developing targeted strategies to mitigate their neurodysfunctional impact.

## Figures and Tables

**Table 1:** Toxicity factors (TF) of PFOA and PFNA under different methods and conditions. TF was calculated as the ratio of the toxic effect (reduction in median survival or AChE activity relative to control) to the corresponding control value.

Methods and Conditions Used to Assess Toxicity	TF_PFOA_	TF_PFNA_
Survival assay, 10% sucrose and 25 °C	22%	45%
Survival assay, 10% sucrose and 29 °C	0%	40%
AChE activity assay (head), 10% sucrose and 25 °C	29%	12%
AChE activity assay (head), 10% sucrose and 29 °C	20%	0%
AChE activity assay (body), 10% sucrose and 29 °C	50%	0%

## Data Availability

The original contributions presented in this study are included in the article. Further inquiries can be directed to the corresponding author.

## Abbreviations

The following abbreviations are used in this manuscript:

PFAS	Per- and polyfluoroalkyl substances
PFOA	Perfluorooctanoic acid
PFNA	Perfluorononanoic acid
AChE	Acetylcholinesterase
ALS	Amyotrophic lateral sclerosis
